# Cross-sectional study of *Mycoplasma hyopharyngis*,* Mycoplasma hyopneumoniae*, *Mycoplasma hyorhinis* and *Mycoplasma hyosynoviae* in the tonsils of fattening pigs from Central-Eastern Europe

**DOI:** 10.1186/s40813-025-00429-6

**Published:** 2025-03-06

**Authors:** Eszter Zsófia Nagy, Dorottya Földi, Fruzsina Madzig, Enikő Wehmann, Adél Orosz, András Kempf, László Buza, János Mátyus, László Búza, Dénes Grózner, Zsuzsa Kreizinger, Miklós Gyuranecz

**Affiliations:** 1 HUN-REN Veterinary Medical Research Institute, Budapest, Hungary; 2 National Laboratory of Infectious Animal Diseases, Antimicrobial Resistance, Veterinary Public Health and Food Chain Safety, Budapest, Hungary; 3 University of Veterinary Medicine, Budapest, Hungary; 4 Bóly Zrt., Bóly, Hungary; 5 Somogy County Government Office, Department of Food Chain Safety and Animal Health, Kaposvár, Hungary; 6 Hungary-Meat Kft., Kiskunfélegyháza, Hungary; 7 MolliScience Kft., Biatorbágy, Hungary

**Keywords:** Cross-sectional study_1_, Fattening pigs_2_, *Mycoplasma hyopharyngis*_3_, *Mycoplasma hyopneumoniae*_*4*_, *Mycoplasma hyorhinis*_5_, *Mycoplasma hyosynoviae*_6_, qPCR_7_, Swine tonsil_8_

## Abstract

**Background:**

*Mycoplasma (M.) hyopharyngis*,* M. hyopneumoniae*,* M. hyorhinis*, and *M. hyosynoviae* can all be transiently present in the swine tonsils without causing any clinical signs or lesions. *M. hyopharyngis* is considered a commensal bacterium, however, our knowledge about its prevalence and pathogenic capabilities is lacking. *M. hyopneumoniae*,* M. hyorhinis* and *M. hyosynoviae* are widespread pathogens, responsible for significant economic losses. *M. hyopneumoniae* is known as the causative agent of porcine enzootic pneumonia, while *M. hyorhinis* and *M. hyosynoviae* are associated with arthritis and polyserositis. The objective of this study was to evaluate the detection rates of these mycoplasmas in Central-Eastern Europe (Croatia, the Czech Republic, Hungary, and Slovakia) through a cross-sectional investigation. In parallel, a novel quantitative polymerase chain reaction (qPCR) assay was designed targeting *M. hyopharyngis* to facilitate the identification of this bacterium.

**Results:**

Tonsils of 15 animals per herd were sampled from six-month-old fattening pigs, and a total of 150 herds were examined. Tonsils form each herd were divided into three pools, each comprising five tonsils. The samples were submitted for species-specific TaqMan assay and isolation. *M. hyopharyngis* was identified in 92.67% (139/150, 95% confidence interval: 87.35–95.86%) of the stocks, with successful isolation from 20 herds. Besides, *M. hyopneumoniae* was detected in 51.33% (77/150, 95% confidence interval: 43.40-59.19%) of the stocks. Additionally, *M. hyorhinis* was identified in all herds (100.00%; 150/150, 95% confidence interval: 97.50–100.00%) by qPCR examination and was successfully isolated from 107 stocks. Regarding the occurrence of *M. hyosynoviae*, 88.00% (132/150, 95% confidence interval: 81.83–92.27) of the herds showed positive PCR results, and the pathogen was successfully isolated in 122 cases. Moreover, the newly developed *M. hyopharyngis* qPCR assay proved to be a reliable and sensitive method.

**Conclusions:**

This study determined the detection rates of several porcine mycoplasmas (*M. hyopharyngis*,* M. hyopneumoniae*,* M. hyorhinis*, and *M. hyosynoviae*) in fattening pigs in Central-Eastern Europe. Additionally, the developed *M. hyopharyngis* qPCR assay may facilitate future prevalence studies and diagnostic procedures concerning this neglected bacterium.

**Supplementary Information:**

The online version contains supplementary material available at 10.1186/s40813-025-00429-6.

## Background

Mycoplasmas are the smallest self-replicating prokaryotes, which are known for the lack of a cell wall as well as the extraordinarily low guanine and cytosine content of their DNA [1]. The swine respiratory tract can be colonized by various *Mycoplasma* species. Besides the most common pathogens, which include *Mycoplasma (M.). hyopneumoniae* [2], *M. hyorhinis*, and *M. hyosynoviae*, other species can also be present that are considered non-pathogenic, such as *M. hyopharyngis* and *M. flocculare* [3].

Four of the most common swine mycoplasmas, including *M. hyopharyngis*,* M. hyopneumoniae*,* M. hyorhinis*, and *M. hyosynoviae*, are the subject of this investigation. Despite their remote phylogenetic relationship and various metabolisms, these porcine mycoplasmas can all be found in the tonsils of swine even without any detectable signs [4–9].

Phylogenetic analysis based on the 16 S rRNA gene places all porcine mycoplasmas within the *M. hominis* group [10, 11]. Additionally, *M. hyopharyngis* belongs to the *M. lipophilum* cluster [10, 11], whereas *M. hyorhinis* and *M. hyopneumoniae* are classified within the *M. neurolyticum* cluster [10–12]. Furthermore, *M. hyosynoviae* is categorized within the *M. hominis* cluster [10, 11]. Moreover, metabolic investigations demonstrated that *M. hyopharyngis* and *M. hyosynoviae* hydrolyze arginine [4, 13], while *M. hyopneumoniae* and *M. hyorhinis* ferment glucose [1].

*M. hyopharyngis* is a commensal mycoplasma, yet knowledge in terms of its prevalence and pathogenic capabilities is lacking [4, 7, 11]. So far, it has been isolated from swine upper respiratory tracts [4], inflamed joints, subcutaneous abscesses [14, 15], and tonsillar surface scrapings [16].

Infection caused by *M. hyopneumoniae* (the causative agent of porcine enzootic pneumonia) results in significant economic losses worldwide due to the expenses associated with medication and the decreased performance of the affected herds [17]. One of the most effective prevention techniques is vaccination, which provides a cost-efficient method to reduce economic losses, clinical signs, and lung lesions resulting from *M. hyopneumoniae* infection [18]. Furthermore, various control strategies have been successfully implemented recently [19]. *M. hyopneumoniae* is primarily transmitted through close contact between infected and susceptible pigs. Clinical signs typically manifest during the grow-finishing stage, with no notable age-related susceptibility. One of the main signs is an intermittent, dry, non-productive cough [20]. The most common diagnostic method for *M. hyopneumoniae* includes quantitative polymerase chain reaction (qPCR), using samples obtained from target tissues such as the bronchi and bronchioles from the lower respiratory tract [20]. The detection of this bacterium from tonsil samples is also feasible [21–23].

*M. hyorhinis* and *M. hyosynoviae* are considered emerging pathogens in the swine industry [24–26]. The diseases caused by these bacterial species are characterized by low mortality and variable morbidity [6, 27]. Due to culling and reduced feed conversion, these pathogens are responsible for significant economic losses [6, 7, 28]. This could be prevented or treated by improving the housing conditions and using antibiotic therapy [7, 29], as currently no vaccines are available commercially against them in Europe [3]. A commercially accessible vaccine for *M. hyorhinis* infections, known as Ingelvac MycoMAX™ (Boehringer Ingelheim Animal Health USA Inc., Duluth, USA), is at present only authorized for use in the United States. However, inadequate antibiotic therapy has led to the development of resistant strains against various antibiotics [30–35].

*M. hyorhinis* is a widespread pathogen that is presumed to be transmitted from dams to piglets through nasal secretions [27]. Septicemia can occur as a result of stress or co-infections, leading to a systemic disease characterized by polyserositis and polyarthritis [27, 36]. Clinical signs typically manifest in pigs aged three to ten weeks, including lethargy, anorexia, lameness, swollen joints, and fever [6, 24, 27]. Although these lesions usually subside after two weeks, lameness with joint swelling may persist for months [6, 24, 27]. Additionally, *M. hyorhinis* infection has been associated with otitis media, eustachitis [37], conjunctivitis [38], and meningitis [39].

Regarding *M. hyosynoviae*, pigs are assumed to become infected by direct oronasal contact [40]. This bacterium tends to colonize the palatine tonsil, being transiently present throughout the host’s lifespan [6, 7, 13]. Clinical signs of *M. hyosynoviae* infection typically appear as swollen joints and lameness in pigs aged 10 to 24 weeks [6, 7, 24].

The detection of *M. hyorhinis* and *M. hyosynoviae* has been described using several sample types, such as PCR examination or bacterial cultivation from nasal swabs, oral or synovial fluids, and tonsil tissue [13, 40–43]. However, a definitive diagnosis often requires samples from joint lesions (in the cases of *M. hyorhinis* and *M. hyosynoviae*) or serosa (specifically for *M. hyorhinis*) [44].

The tonsil is known to be a site of colonization for many bacteria, including porcine mycoplasmas [45, 46]. It has been identified as an appropriate location to evaluate the presence of *M. hyosynoviae* [13], and other porcine mycoplasmas, such as *M. hyopharyngis*, *M. hyopneumoniae* and *M. hyorhinis* [13, 21–23, 40, 42, 43]. Given these characteristics and the fact that these mycoplasmas can inhabit the tonsils without causing clinical signs [4–9], the current research focused on the examination of samples from this tissue.

This study represents the first comprehensive investigation of the detection rates of these four swine *Mycoplasma* species in Central-Eastern Europe using cross-sectional sampling of tonsil samples. Furthermore, this information contributes to the knowledge of their occurrence and outlines the importance of implementing control methods.

## Methods

### Origin of tonsils

The examined tonsils (tonsils of the soft palate) were collected from apparently healthy, approximately six-month-old fattening pigs from a total of 150 different herds at slaughter. These observed herds are located in Hungary and three neighboring countries (Croatia, the Czech Republic, and Slovakia; Additional Tables 1 and 2). According to the written declaration (reference number: VMRI/2022/0021) of the Ethics Committee of the Veterinary Medical Research Institute ethical approval was not required for the study as the samples were taken during the slaughter process with the written consent of the owner. The collected 2250 tonsils (15 tonsils per herd, from different animals) were stored at -70 °C. Power analysis was conducted to determine the required sample size for detecting effect sizes of 20% (small), 50% (medium), and 80% (large), based on Cohen’s (1982) definitions [47]. The calculation was carried out by R software version 4.3.3 [48].

**Table 1:** Primer sequences and characteristics of the 16 S rRNA targeting qPCR

Primer	Sequence (5’ → 3’)	Gene position*	Melting temperature	GC content**
**Forward**	AGGTTGTTTATTAAGTCTGACGTT	573–596	56.1 °C	33.3%
**Reverse**	CGCTTCACAAGGAATTCCG	661–679	56.7 °C	52.6%
**Probe**	FAM-CCCAACCCGCGTTAGATACTGG-BHQ1	614–635	62.6 °C	59.1%

*numbered according to 16 S rRNA sequences in the GenBank; Accession numbers: U58997.1 and NR_037123.1

**GC: guanidine – cytosine

**Table 2:** Taqman-type qPCR and isolation results of the examined Mycoplasmas

			qPCR quantification cycles (C_q_)				
			Negative	35–40	30–35	25–30	20–25
***M. hyopharyngis***	**Negative**	**qPCR**	11				
	**1 positive pool**	**qPCR**		3	2		
		**Isolation**		0	0		
	**2 positive pools**	**qPCR**		6	4	2	
		**Isolation**		0	0	0	
	**3 positive pools**	**qPCR**		10	51	61	
		**Isolation**		0	6	14	
***M. hyopneumoniae***	**Negative**		73				
	**1 positive pool**	**qPCR**		14	1	1	1
	**2 positive pools**			4	4	1	
	**3 positive pools**			10	26	15	
***M. hyorhinis***	**Negative**	**qPCR**					
	**1 positive pool**	**qPCR**		1	1	6	1
		**Isolation**		0	1	3	1
	**2 positive pools**	**qPCR**		3	19	18	4
		**Isolation**		3	13	14	3
	**3 positive pools**	**qPCR**		1	21	70	5
		**Isolation**		1	11	52	5
***M. hyosynoviae***	**Negative**	**qPCR**	18*				
	**1 positive pool**	**qPCR**		5	6	4	1
		**Isolation**		1	4	4	1
	**2 positive pools**	**qPCR**		5	12	18	3
		**Isolation**		5	10	17	3
	**3 positive pools**	**qPCR**		1	22	45	10
		**Isolation**		1	20	45	10

The table displays the number of positive herds, categorized by the number of positive pools, along with their mean Cq values within individual herds. It also shows the number of successful isolations within each group. *M. hyopneumoniae* isolation was not conducted in this study

*In the case of *M. hyosynoviae*, the C_q_ of one stock was above the cut-off value (C_q_=40.66), so it was considered negative, although the isolation was successful

### DNA extraction

The 15 tonsils from each herd were divided into three pools and subjected to homogenization in phosphate buffered saline using a BagMixer 400 ml (Interscience, Saint-Nom-la-Bretèche, France) for 8 min at the speed of 4 strokes/s. One milliliter of each homogenate was centrifuged for 9 min at 9000 × g, and the supernatant was discarded. Subsequently, DNA extraction was performed according to the manufacturer’s instructions with the ReliaPrep™ gDNA Tissue Miniprep System (Promega Inc., Madison, Wisconsin, USA).

### Introduction of the applied qPCR assays and the newly developed *M. hyopharyngis* specific TaqMan qPCR

The species-specific TaqMan-type qPCR assays targeting *M. hyorhinis* [49], and *M. hyosynoviae* [50] were designed to amplify the 16 S rRNA gene, while the target region of the *M. hyopneumoniae* qPCR assay is the p97 coding gene [51]. The primers and probe for the *M. hyopharyngis* qPCR assay were designed using the GeneScript TaqMan primers and probe design tool [52], followed by subsequent manual optimization. The design was based on two available 16 S rRNA gene sequences from the NCBI database: GenBank Acc. No. U58997.1 (*Mycoplasma hyopharyngis* 16 S ribosomal RNA gene, 1454 bp [53]), and NR_037123.1 (*Mycoplasmopsis hyopharyngis* strain H3-6B 16 S ribosomal RNA, partial sequence, 1454 bp [54]),. Primer sequences, gene positions, melting temperatures, and guanidine-cytosine contents are detailed in Table 1. The size of the PCR amplicon is 107 base pairs. The used Mastermix compositions and PCR protocol are provided in Additional Table 3. The PCR reactions were carried out using the CFX96 Touch real-time PCR Detection System (Bio-Rad Laboratories, Watford, USA).

**Table 3:** The detection rates of swine Mycoplasmas across different countries

Country	Number of examined herds	PCR (percentage and number of positive pools/ total number of herds)				Isolation (percentage and number of positive pools/ total number of herds)		
		*M. hyopharyngis*	*M. hyopneumoniae*	*M. hyorhinis*	*M. hyosynoviae*	*M. hyopharyngis*	*M. hyorhinis*	*M. hyosynoviae*
Croatia	8	100.00% (8/8)	37.50% (3/8)	100.00% (8/8)	87.50% (7/8)	0.00% (0/8)	87.50% (7/8)	87.50% (7/8)
Czech Republic	5	100.00% (5/5)	40.00% (2/5)	100.00% (5/5)	100.00% (5/5)	20.00% (1/5)	80.00% (4/5)	100.00% (5/5)
Hungary	134	91.79% (123/134)	52.24% (70/134)	100.00% (134/134)	88.80% (119/134)	14.18% (19/134)	70.15% (94/134)	81.34% (109/134)
Slovakia	3	100.00% (3/3)	66.67% (2/3)	100.00% (3/3)	33.33% (1/3)	0.00% (0/3)	66.67% (2/3)	33.33% (1/3)

In this study, *M. hyopneumoniae* isolation was not carried out

### Validation of the novel *M. hyopharyngis* specific qPCR

For the development and validation of the *M. hyopharyngis* TaqMan qPCR system, a gBlock™ gene fragment (Integrated DNA Technologies Inc., Coralville, Iowa, USA) covering the PCR amplicon was purchased (the sequence of the gBlock is provided in Additional Table 4). To evaluate the sensitivity and repeatability of our PCR assay, ten-fold dilution series were made from the *M. hyopharyngis* specific synthetic DNA (initial concentration in Additional Table 5) in the range of 10^6^ to 10^1^ template copy numbers/µl. For analysis, each dilution point was evaluated six times on a single plate, following the guidelines outlined in a previous study [55]. To ensure robustness, three independent experimental runs were conducted, resulting in a total of 18 data points per dilution level [55].

**Table 4:** Coinfections found in the herds under investigation

Coinfections and their occurrence in percentage detected by PCR (number of the PCR positive herds / all herds)			Percentage of herds with successful isolation (number of herds with successful isolation/PCR positive herds)					
		*M. hyopharyngis*, *M. hyorhinis* and *M. hyosynoviae*		*M. hyorhinis and M. hyosynoviae*	*M. hyopharyngis and M. hyosynoviae*	*M. hyorhinis*	*M. hyosynoviae*	None
*M. hyopharyngis*,* M. hyopneumoniae*,* M. hyorhinis* and *M. hyosynoviae*	42.00% (63/150)	7.94% (5/63)		60.32% (38/63)	4.76% (3/63)	3.17% (2/63)	23.80% (15/63)	
*M. hyopharyngis*,* M. hyorhinis* and *M. hyosynoviae*	41.33% (62/150)	9.68% (6/62)		45.16% (28/62)	8.06% (5/62)	11.29% (7/62)	24.19% (15/62)	1.61% (1/62)
*M. hyopharyngis*,* M. hyopneumoniae* and *M. hyorhinis*	5.33% (8/150)					87.50% (7/8)		12.50% (1/8)
*M. hyopneumoniae*,* M. hyorhinis* and *M. hyosynoviae*	2.67% (4/150)			25.00% (1/4)		25.00% (1/4)	50.00% (2/4)	
*M. hyorhinis* and *M. hyosynoviae*	2.00% (3/150)			100% (3/3)				
*M. hyopharyngis* and *M. hyorhinis*	4.00% (6/150)				16.67% (1/6)	83.33% (5/6)		
*M. hyopneumoniae* and *M. hyorhinis*	1.33% (2/150)					100.00% (2/2)		

Sole *M. hyopharyngis* or *M. hyosynoviae* infection was not detected by PCR, and there was no precedent for the isolation of *M. hyopharyngis* alone. In this study *M. hyopneumoniae* isolation was not carried out

In order to determine the diagnostic sensitivity of *M. hyopneumoniae*, *M. hyorhinis*, and *M. hyosynoviae* qPCR assays, ten-fold dilution series of *Mycoplasma hyopneumoniae* NCTC 10,110, *Mycoplasma hyorhinis* NCTC 10,130, and *Mycoplasma hyosynoviae* NCTC 10,167 type strains were prepared and evaluated six times on a single plate as described above.

The in vitro specificity assessment of the *M. hyopharyngis* qPCR assay involved the utilization of the type strains of porcine mycoplasmas, namely *M. hyorhinis* (NCTC 10130), *M. hyosynoviae* (NCTC 10167), *M. hyopneumoniae* (NCTC 10110), and *M. flocculare* (NCTC 10143). Genomic DNA was extracted from logarithmic phase culture using a commercial kit (QIAamp DNA Mini Kit, Qiagen Inc., Hilden, Germany) following the manufacturer’s instructions. For the assessment, the porcine type strains (Additional Table 5) were individually tested, as well as in combination with *M. hyopharyngis* DNA. In the latter case, the genome copy number/µl of *M. hyorhinis*,* M. hyosynoviae*,* M. hyopneumoniae*, and *M. flocculare* ranged from 10^2^ to 10^5^, while the template copy number of the *M. hyopharyngis* specific synthetic DNA remained constant at 10^2^/µl.

In addition, a total of 20 pathogenic and non-pathogenic bacterial strains commonly found in swine tonsils were examined for potential cross-reactions. Strains of the following species were included in the tests: *Actinobacillus pleuropneumoniae*,* Actinobacillus suis*,* Bacillus cereus*,* Bordetella bronchiseptica*,* Clostridium perfringens*,* Erysipelothrix rhusiopathiae*,* Escherichia coli*,* Glaeserella parasuis*,* Listeria monocytogenes*,* Pasteurella multocida*,* Proteus mirabilis*,* Rhodococcus equi*,* Salmonella enterica*,* Staphylococcus aureus*,* Staphylococcus hyicus*,* Streptococcus porcinus*,* Streptococcus suis*,* Trueperella pyogenes*,* Yersinia enterocolitica* and *Yersinia pseudotuberculosis* (Additional Table 5). The DNA extraction was performed as described above.

The results of a conventional PCR specific for porcine mycoplasmas [56] and the developed *M. hyopharyngis* TaqMan qPCR assay were compared using the DNA extracted from tonsils originating from slaughtered pigs from 20 different herds in Hungary. The conventional PCR was considered positive for *M. hyopharyngis* when a 360 bp long product was present [56] and in this case, no sequencing was performed for definitive species identification.

Data analysis was performed using the Bio-Rad CXF Maestro 1.1 software (version 4.1.2433.1219, Bio-Rad Laboratories). The most appropriate cutoff quantification cycle (C_q_) value was calculated using Youden’s index [57].

### Culture of mycoplasmas

Each homogenate was inoculated into MolliScience General Mycoplasma (GM) liquid media (MolliScience Kft., Biatorbágy, Hungary) and this suspension was filtered through a 0.45 μm filter (VWR^®^ syringe filters, VWR International LLC., Radnor, Pennsylvania, USA). A tenfold sub-dilution was then performed in MolliScience GM liquid media. Following this, the broths were incubated at 37 °C, and 5 µl droplets from both the original broth and the sub-dilutions were inoculated onto MolliScience GM solid media (MolliScience Kft.) on the initial day and subsequently every three days. Additionally, streak cultures were made on MolliScience GM solid media (MolliScience Kft.) upon detection of color changes in the broth. The cultures on solid media were incubated at 37 °C with a 5% CO_2_ supply and typically yielded colonies within 2–5 days. Mycoplasma-like single colonies (approximately 5 colonies/sample) were picked into sterile MolliScience GM liquid media and then incubated at 37 °C until detectable color changes. Identification of the pure cultures was performed by DNA extraction and the species-specific TaqMan qPCR as described above. *M. hyopneumoniae* isolation was not carried out.

### Statistical analysis

The apparent and true detection rates were calculated using the Bayesian method [58–60] in R software version 4.3.3 [48]. Regression analysis was used to ascertain whether coinfections (determined by qPCR), the number of positive pools, or the mean C_q_ value of the positive pools within one herd could have an impact on the successful isolation of each pathogen. Regression and power analysis were performed using R software version 4.3.3 [48].

## Results

Regarding *M. hyopharyngis* qPCR validation, all examined values to characterize the efficiency (correlation coefficient, amplification efficiency, slope values, y-intercept) and repeatability (standard deviation of the quantification cycles and coefficients of variability) of the developed qPCR were found to be in the acceptable ranges [55, 61] and are detailed in Additional Table 6. Comparing the developed *M. hyopharyngis* qPCR and conventional PCR methods, the former enabled the identification of *M. hyopharyngis* in all samples, while products in the target size could be observed only in five cases with conventional PCR (Additional Table 7). Moreover, the qPCR method was proven to be specific to the target bacterium, with no observed false positive results.

The lowest detectable DNA concentration was 10^1^ copies/µl for each qPCR system in this study. The diagnostic sensitivity values were 0.878 for the *M. hyopharyngis* qPCR, 0.972 for the *M. hyopneumoniae* qPCR, 0.923 for the *M. hyorhinis* qPCR, and 0.972 for the *M. hyosynoviae* qPCR. The cutoff values calculated using Youden’s index were 40 C_q_ for each qPCR assay. This value was applied during the determination of the number of positive pools and in the comparison of positive qPCR results and successful isolations as well. The background information for the applied qPCR assays is provided in Additional Tables 6 and 8.

Regarding the results of the power analysis, the study is well-powered to detect medium and large effect sizes with high probability. Even for the small effect size, the study shows a moderate detection probability of 68.78%.

**Fig. 1:**
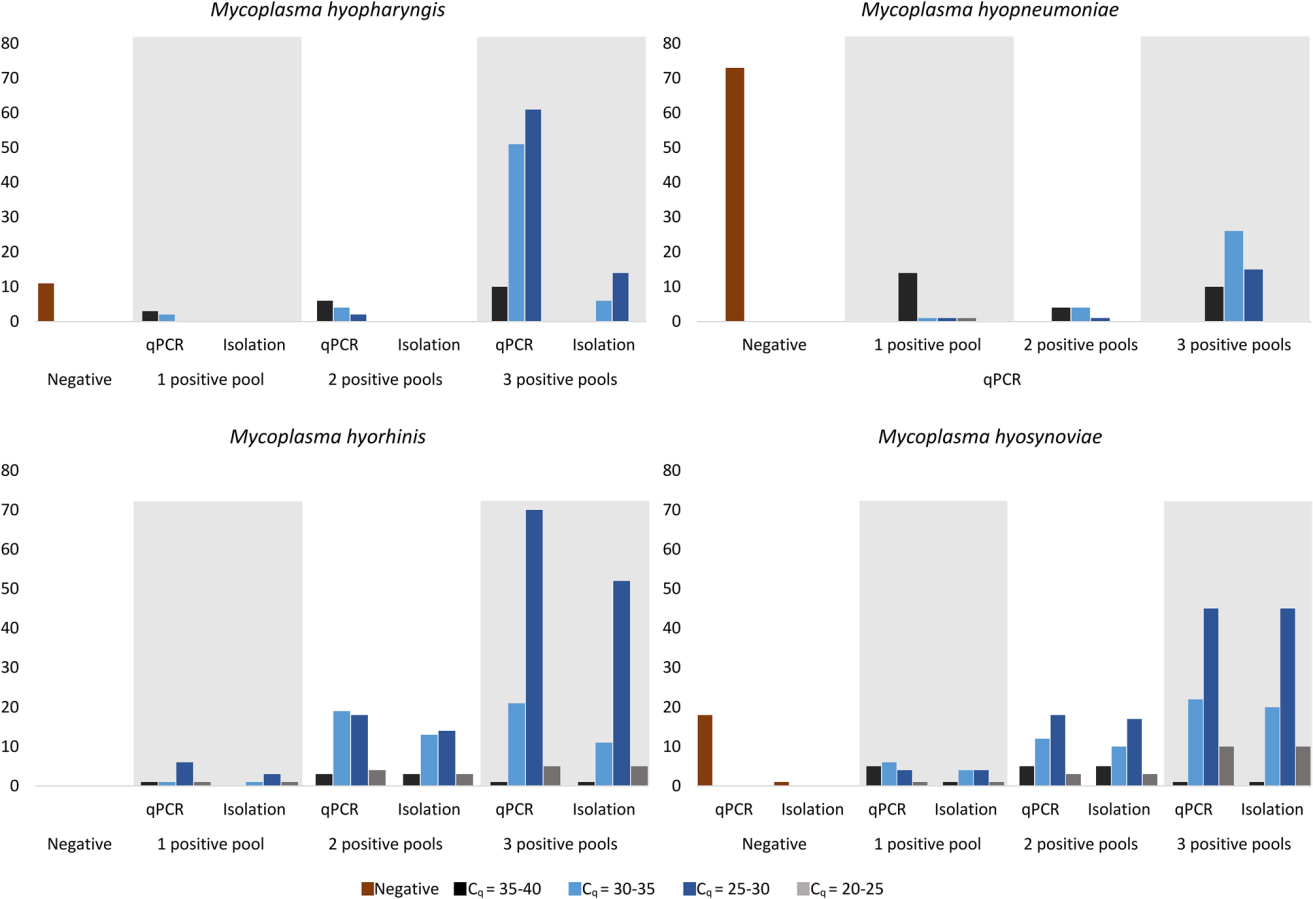
Taqman-type qPCR and isolation results of *Mycoplasma (M.) hyopharyngis* (**A**), *M. hyopneumoniae* (**B**), *M. hyorhinis* (**C**), and *M. hyosynoviae* (**D**). In the chart, the positive herds are represented by the number of positive pools and their mean C_q_ values within each stock. Furthermore, the chart illustrates how many isolations were successful within each group based on the qPCR results. Additionally, in the case of *M. hyosynoviae*, the C_q_ of one stock was above the cut-off (C_q_=40.66), thus it was considered negative, although the isolation was successful

The observed percentage of qPCR-positive herds for the presence of *M. hyopharyngis* was 92.67% (139/150, 95% confidence interval: 87.35–95.86%), while the estimated true detection rate of qPCR-positive herds was 98.7% (95% credible interval: 95.3–100.0%). The distributions of detection levels are detailed in Additional Tables 1 and 2, as well as summarized in Table 2; Fig. 1/A. The bacterium was successfully isolated from 20 herds, all exhibiting three PCR-positive pools (Additional Tables 1, 2 and 2; Fig. 1/A).

Figure 1. Taqman-type qPCR and isolation results of *Mycoplasma hyopharyngis* **(A)**, ***M. hyopneumoniae*** **(B)**, ***M. hyorhinis*** **(C)**, and ***M. hyosynoviae*** **(D).** In the chart, positive herds are represented by the number of positive pools and their mean C_q_ values within each stock. Furthermore, the chart illustrates how many isolations were successful within each group based on the qPCR results. Additionally, in the case of *M. hyosynoviae*, the C_q_ of one stock was above the cut-off value (C_q_=40.66), thus it was considered negative, although the isolation was successful. In this study, *M. hyopneumoniae* isolation was not carried out.

Table 2. Taqman-type qPCR and isolation results of the examined mycoplasmas. The table displays the number of positive herds, categorized by the number of positive pools, along with their mean C_q_ values within individual herds. It also shows the number of successful isolations within each group. Additionally, in the case of *M. hyosynoviae*, the C_q_ of one stock was above the cut-off value (C_q_=40.66), thus it was considered negative, although the isolation was successful. *M. hyopneumoniae* isolation was not conducted in this study.

The observed percentage of *M. hyopneumoniae* presence was 51.33% (77/150, 95% confidence interval: 43.40-59.19%), with an estimated true detection rate of 52.7% (95% credible interval: 44.4–60.8%) (Additional Tables 1, 2 and 2; Fig. 1/B).

Regarding *M. hyorhinis*, all stocks (100.00%; 150/150, 95% confidence interval: 97.50–100.00%) tested positive, with an estimated true incidence rate of 99.4% (95% credible interval: 98.1–100.0%) (Additional Tables 1, 2 and 2; Fig. 1/C). Isolation of *M. hyorhinis* was successful in 107 herds, revealing single, double, or triple positive pools with a broad range of Cq values (see Additional Tables 1, 2 and 2; Fig. 1/C).

For *M. hyosynoviae*, 88.00% of the herds tested positive (132/150, 95% confidence interval: 81.83–92.27), with an estimated true detection rate of 83.2% (95% credible interval: 76.3–89.2%) (Additional Tables 1, 2 and 2; Fig. 1/D). The isolation of *M. hyosynoviae* was achieved in 122 herds with single, double and triple PCR positive pools of all detected C_q_ ranges. In one stock, although the C_q_ of the *M. hyosynoviae* PCR was above the cut-off value (C_q_ = 40.66) and thus considered negative for this pathogen, the isolation of this bacterium was successful.

In terms of different countries (Table 3), varying occurrence rates were observed for *M. hyopharyngis*,* M. hyopneumoniae*,* M. hyorhinis*, and *M. hyosynoviae* in the examined herds. In Croatia, all groups (100.00%, 8/8) exhibited positivity for *M. hyopharyngis* and *M. hyorhinis*, while three were positive (37.50%, 3/8) for *M. hyopneumoniae*, and seven herds (87.50%, 7/8) tested positive for *M. hyosynoviae* during qPCR examination. In the Czech Republic, all stocks tested positive for *M. hyopharyngis*, *M. hyorhinis*, as well as *M. hyosynoviae* (100.00%, 5/5), and two herds (66.67%, 2/3) were positive for *M. hyopneumoniae* using qPCR analysis. In Hungary, a significant proportion of groups were positive for *M. hyopharyngis* (91.79%, 123/134), *M. hyopneumoniae* (52.24%, 70/134), *M. hyorhinis* (100.00%, 134/134), and *M. hyosynoviae* (88.80%, 119/134) based on qPCR examination. In Slovakia, qPCR examination identified *M. hyopharyngis*, and *M. hyorhinis* in all groups (100.00%, 3/3), while *M. hyopneumoniae* was identified in 66.6% (2/3) and *M. hyosynoviae* was detected in 33.33% (1/3) of the groups. The results of the subsequent isolation can be seen in Table 3.

Table 3. The detection rates of swine mycoplasmas across different countries. In this study, *M. hyopneumoniae* isolation was not carried out.

The observed coinfections within individual herds are listed in Table 4. The qPCR examination revealed the simultaneous presence of all examined bacteria in 42.00% (63/150) of the stocks. Concurrent infection by three different bacteria was observed in 41.33% (62/150) of the herds (*M. hyopharyngis*, *M. hyorhinis*,* M. hyosynoviae*) and in 5.33% (8/150) of the herds (*M. hyopharyngis*,* M. hyopneumoniae*,* M. hyorhinis*). Additionally, coinfections involving two mycoplasmas were identified in 2.00% (3/150) (*M. hyorhinis* and *M. hyosynoviae*), 4.00% (6/150) (*M. hyopharyngis* and *M. hyorhinis*), and 1.33% (2/150) (*M. hyopneumoniae* and *M. hyorhinis*) of the stocks. The successful isolations from these samples are detailed in Table 4.

Table 4. Coinfections found in the herds under investigation. Sole *M. hyopharyngis* or *M. hyosynoviae* infection was not detected by qPCR, and there was no precedent for the isolation of *M. hyopharyngis* alone. In this study, *M. hyopneumoniae* isolation was not carried out.

Additionally, a regression analysis was constructed to investigate factors (coinfections, number of qPCR positive pools within individual herds, and their mean C_q_ value) influencing the success of the bacteria culture. Results indicate statistically significant correlations between the successful isolation of *M. hyosynoviae* and the number or mean C_q_ values of the positive pools within individual herds (*p* = 0.004 and *p* = 0.001, respectively), while coinfections exhibited no significance (*p* = 0.109). However, the model’s overall explanatory power is limited (R-squared = 0.256). Additionally, results reveal a significant association between the successful isolation of *M. hyorhinis* and the mean C_q_ value of the positive pools within one herd (*p* = 0.019), while coinfections and the number of positive pools showed non-significant relationships. Nevertheless, this model also demonstrates limited explanatory power (R-squared = 0.038). In the case of *M. hyopharyngis* isolation, neither coinfections, the number of positive pools, nor the mean C_q_ value demonstrated significant relationships. The regression analysis demonstrates a statistical power of 0.882.

## Discussion

This investigation represents a cross-sectional study focusing on four swine *Mycoplasma* species in Hungary and three neighboring countries (Croatia, the Czech Republic, and Slovakia). The main goal of this study was to detect these porcine mycoplasmas in tonsils obtained from slaughtered fattening pigs. These pathogens can occur at this sampling site in healthy pigs without causing any clinical signs [3–7, 9]. Definitive diagnosis of these pathogens can be achieved by obtaining samples from affected joints and serous membranes in the case of *M. hyorhinis* or by aseptically collecting synovia from affected joints regarding *M. hyorhinis* and *M. hyosynoviae* [11]. The diagnosis of *M. hyopneumoniae* can be challenging because of its chronic nature and affinity for epithelial cells in the lower respiratory tract [11, 62]. The appropriate diagnostic approach should be selected based on the diagnostic purpose, whether it involves identifying respiratory diseases or lesions, detecting subclinical infections in pigs within eradication initiatives, or ascertaining the absence of infection [11].

During this study, *M. hyopharyngis* was successfully isolated from 20 herds. Regarding the fact that *M. hyopharyngis* is often described as a non-pathogenic commensal, limited information is available about the distribution of this bacterium and the frequency of the infection [7]. Previous studies have reported low isolation rates in the United States [4], the United Kingdom [14], Denmark [16], Switzerland, and Germany [44]. These isolation frequencies align with our results. On the other hand, our PCR examination revealed an exceptionally high detection rate of *M. hyopharyngis* (92.67%, 139/150, 95% confidence interval: 87.35–95.86%). The lack of prevalence studies focusing on this bacterium contributes to the novelty of the observed high detection rate of this microbe.

Regarding *M. hyopneumoniae* detection, 51.33% (77/150, 95% confidence interval: 43.40-59.19%) of the herds proved to be positive in our examination. It is assumed that this relatively lower prevalence is the results of the eradication programs undertaken in the studied countries.

In terms of *M. hyorhinis*, the pathogen was present in all examined herds (100.00%, 150/150, 95% confidence interval: 97.50–100.00%) with varying levels of detection. Additionally, successful isolation of the pathogen was achieved in 71.3% (107/150) of the affected herds. This bacterium is a common inhabitant of the swine upper respiratory tract, and its presence in swine herds has been reported worldwide. In a similar study conducted in the United States, oral fluid samples were collected from approximately 450 pigs (aged 3–28 weeks) per herd by using ropes. The samples were examined applying qPCR, and the reported presence of the bacteria was comparable to our findings [42]: *M. hyorhinis* was detected in all age groups across four states in the US, namely Minnesota, South Dakota, Wisconsin, and Nebraska. Furthermore, only one herd with six-week-old pigs tested negative in Iowa State out of the seven investigated groups.

Several studies conducted in the US have explored the prevalence of *M. hyorhinis* by qPCR across different age groups of swine. In one study, the prevalence of this pathogen in tonsillar swabs was relatively high in dams at one- and three-weeks post-farrowing (40%). Additionally, the number of positive cases among piglets increased from 8.3 to 50% during a three-week period [46]. Moreover, Clavijo and co-workers conducted PCR examinations on nasal swabs obtained from piglets, growing pigs, and sows in the US in 2017 and 2019 [62, 63]. Their findings indicated that the prevalence of *M. hyorhinis* was low in sows in both investigations (the distribution of the positive dams was < 8% [62] and < 4.7% [63]). In contrast, post-weaning pigs exhibited extremely high percentages (≥ 98%) of *M. hyorhinis* positivity in both examinations [62, 63].

Several published studies have investigated the prevalence of *M. hyorhinis* using lung samples or pleural, peritoneal, and pericardial swabs obtained from pigs at different ages with respiratory disease or polyserositis. Among these studies, the highest reported prevalence was in Taiwan, where PCR examination of lung samples yielded a detection rate of 99.4% [25]. The most common detection rate was approximately 40–60% [64–67], while *M. hyorhinis* exhibited a lower frequency (10–20%) in Switzerland and Germany using PCR examination [44].

Regarding *M. hyosynoviae*, our study detected the pathogen in 88.00% (132/150, 95% confidence interval: 81.83–92.27) of the herds, and successful cultivation was achieved in 92.4% (122/132) of the PCR-positive stocks. High detection rates (70% of the groups) were observed as well, when oral swabs from 10 to 28 weeks old pigs in the US were examined using qPCR [42]. Another study surveyed the prevalence of *M. hyosynoviae* using PCR examination on oral fluid samples from 17 farms across Iowa, South Dakota, Wisconsin, and Nebraska [68], reporting a relatively high prevalence of *M. hyosynoviae* with 52.94% positive farms.

Several studies have surveyed the occurrence of *M. hyosynoviae* infection in swine of different ages using tonsil samples. In Denmark, tonsillar scrapings from three herds were examined by cultivation [69]: the initial minimal infection rate in two herds ranged from 0 to 9%, which increased to 69–86% at 14–15 weeks and exhibited high detection rates at the time of slaughter (88–100%), while one herd remained negative [69]. In terms of detection patterns in dams and piglets, PCR examination of tonsillar swabs showed positive results in 40% of dams after the first to third weeks post-farrowing; however, the distribution was minimal among piglets (0-0.9%) [46]. Successful isolation was reported from 322/400 tonsil samples (80.5% detection rate), with significantly higher isolation rates from pigs with lameness compared to animals without clinical signs [70]. *M. hyosynoviae* prevalence was lower when the pathogen was isolated using synovial fluid from lame (20%) and healthy (8%) grower-finisher pigs in Denmark [71]. In another study, non-purulent arthritis in Danish slaughter pigs was investigated, and *M. hyosynoviae* was isolated from 9% of the joints [72].

The varying detection rates observed can be attributed to differences in animal housing, age, medical history, and the types of samples analyzed. In our study, as a result of PCR examinations, at least one porcine mycoplasma species could be identified in every herd, and successful isolation was achieved with high rates for *M. hyorhinis* (71.3%) and *M. hyosynoviae* (92.4%). Interestingly, cultivation from tonsils appeared to favor *M. hyosynoviae*, as this mycoplasma could be isolated with the highest frequency (92.4% of the PCR positive herds). In a previous study focusing on Danish herds with high lameness incidences, a high rate of successful cultivation using tonsil tissue (75.3%) was reported, in contrast to synovial fluid (~ 10%) or tonsil scrapings (65.9%) [71]. Additionally, another previous research highlighted the examination of tonsils as the most effective method for detecting *M. hyorhinis* and *M. hyosynoviae* infections in asymptomatic cases [73]. These findings are consistent with the higher detection rates observed in our study in relation to these pathogens. Although a robust comparison could not be performed because we examined tonsils from only a few herds from neighboring countries, similar distributions of swine mycoplasmas were observed compared to the results from herds in Hungary. In terms of the predictive factors associated with successful isolation, the statistical analysis revealed that the co-presence of multiple *Mycoplasma* species did not show an inhibitory effect on the bacterial growth in this study. In the cases of *M. hyorhinis* and *M. hyosynoviae*, C_q_ value was one of the main predictive factors, although the models’ overall explanatory powers were limited, suggesting that additional variables may be relevant for a more comprehensive understanding. In terms of *M. hyopharyngis* isolation, it was successful only in the cases of herds with three positive pools and C_q_ value between 25 and 35, although the statistical calculations did not show any correlation. However, these results can vary based on the applied broth and cultivation method. Additionally, samples from animals that developed clinical signs may also show different rates of isolation. Unfortunately, the examined tonsils in this study were collected in slaughterhouses from apparently healthy pigs, and data about possible lesions observed during necropsy are not available.

In summary, our investigation reveals notably high detection rates of the four examined porcine mycoplasmas (*M. hyopharyngis*,* M. hyopneumoniae*,* M. hyorhinis*, and *M. hyosynoviae*) in Hungary. Although *M. hyopharyngis* is considered apathogenic, it has been identified in different lesions [4, 14–16]. By evaluating its detection rate and developing a novel qPCR assay, the current study can contribute to the knowledge about this less-examined bacterium. Regarding other observed porcine mycoplasmas, our examinations support previous studies, which have highlighted the emerging occurrence of *M. hyorhinis* and *M. hyosynoviae* [24, 74].

In terms of control methods, various eradication protocols are applied around the world against *M. hyopneumoniae*. These mainly include repopulation and depopulation, partial depopulation (Swiss method), herd closure and medication, as well as whole herd medication without herd closure [11]. Owing to eradication programs, the occurrence of *M. hyopneumoniae* could be successfully decreased in some countries, including Switzerland [75, 76], Norway [77], Finland [78], and North America [79] and probably in countries included in the present study.

Control of *M. hyosynoviae* and *M. hyorhinis* poses challenges due to their commensal characteristics [80] and the multifactorial aspects underlying associated illnesses [5, 81]. In the case of *M. hyosynoviae*, control methods include the application of autogenous vaccines, but field data are limited [82] or report unsuccessful results [83]. Besides one commercial vaccine against *M. hyorhinis* (Ingelvac MycoMAX™, Boehringer Ingelheim Animal Health USA Inc., Duluth, USA), control methods can include autogenous vaccines [36], as well as an effective experimental inactivated vaccine [84, 85].

Considering limited information on the occurrence of porcine mycoplasmas in this area, this study contributes to understanding the detection rate of these pathogens in fattening swine populations. This information can support practitioners and swine producers in making decisions regarding control strategies [64]. Additionally, the newly developed TaqMan qPCR assay provides a sensitive, convenient, and time-efficient system, valuable for enhancing our understanding of the epidemiology and pathogenic potential of *M. hyopharyngis.*

## Conclusion

Our findings can contribute to broadening our knowledge about the detection rates of *M. hyopharyngis*,* M. hyopneumoniae*,* M. hyorhinis*, and *M. hyosynoviae* in this region. Furthermore, the developed *M. hyopharyngis* qPCR assay may support future prevalence studies and the diagnosis of this minor pathogen.

## Electronic supplementary material

Below is the link to the electronic supplementary material.

Supplementary Material 1

## Data Availability

All data generated or analysed during this study are included in this published article [and its supplementary information files].

## Abbreviations

C_q_: quantification cycles
M: Mycoplasma
qPCR: quantitative polymerase chain reaction
